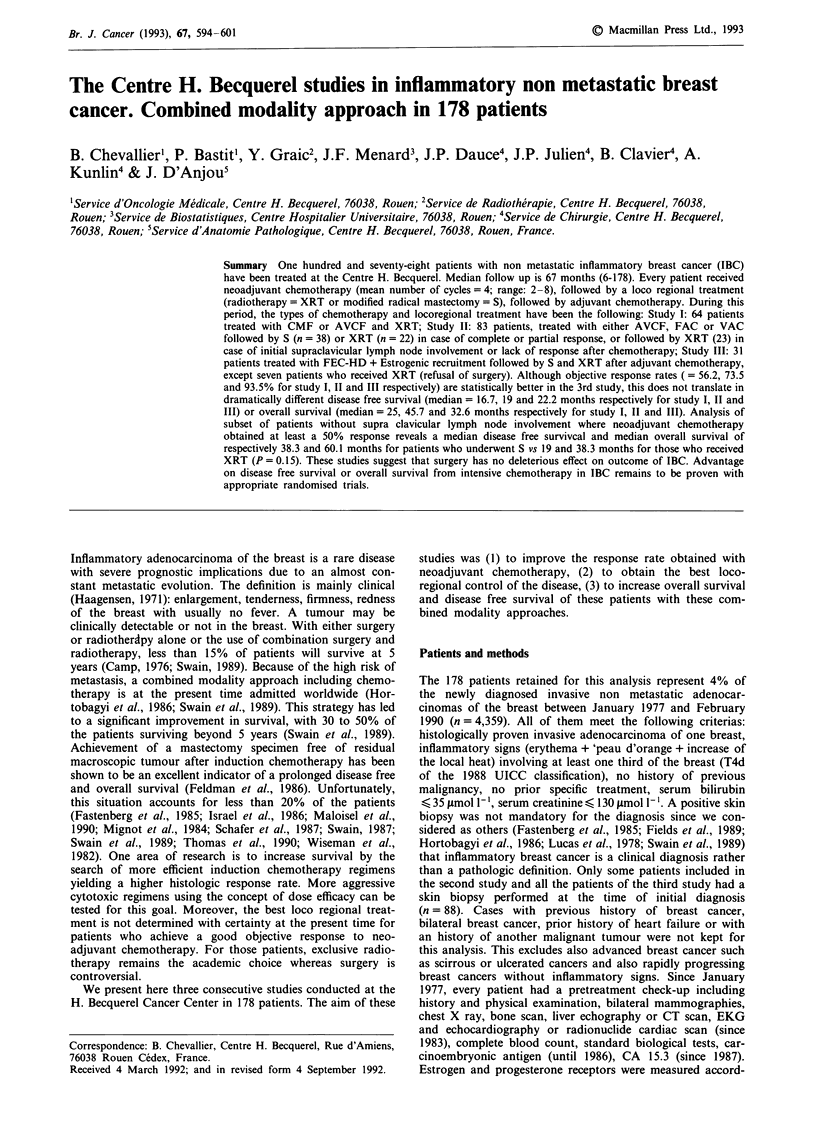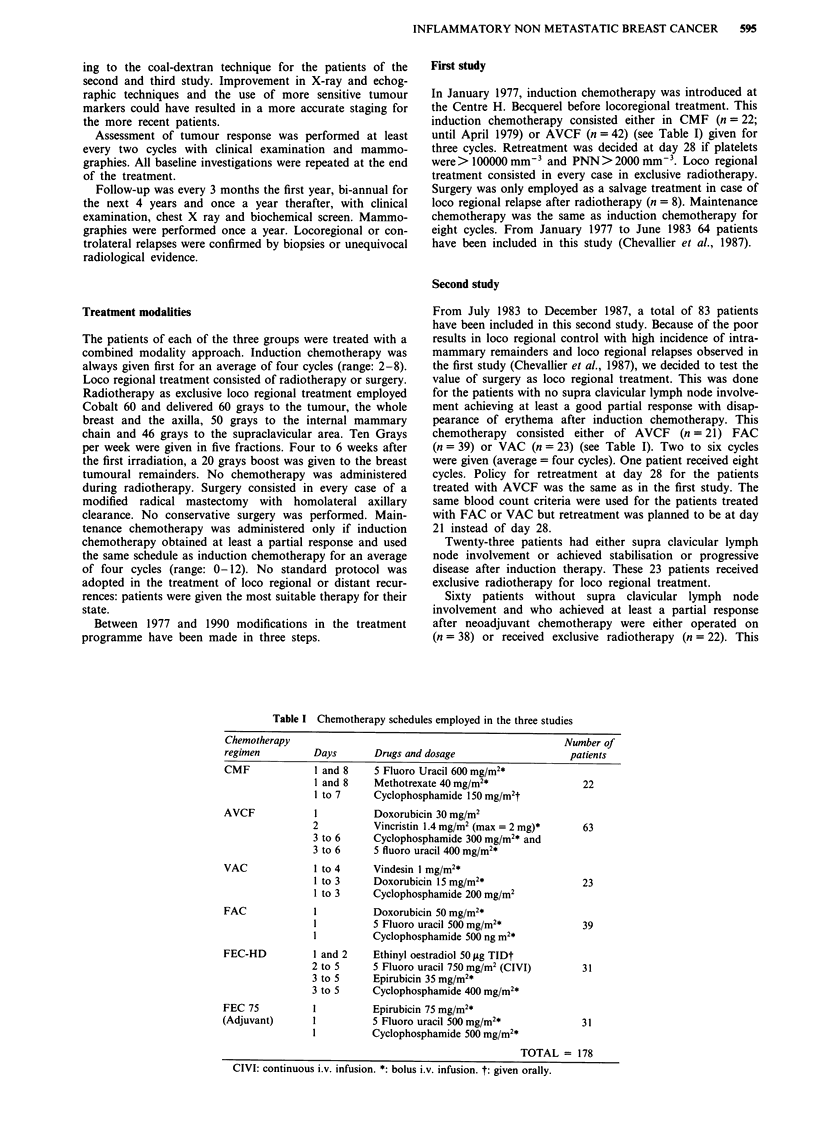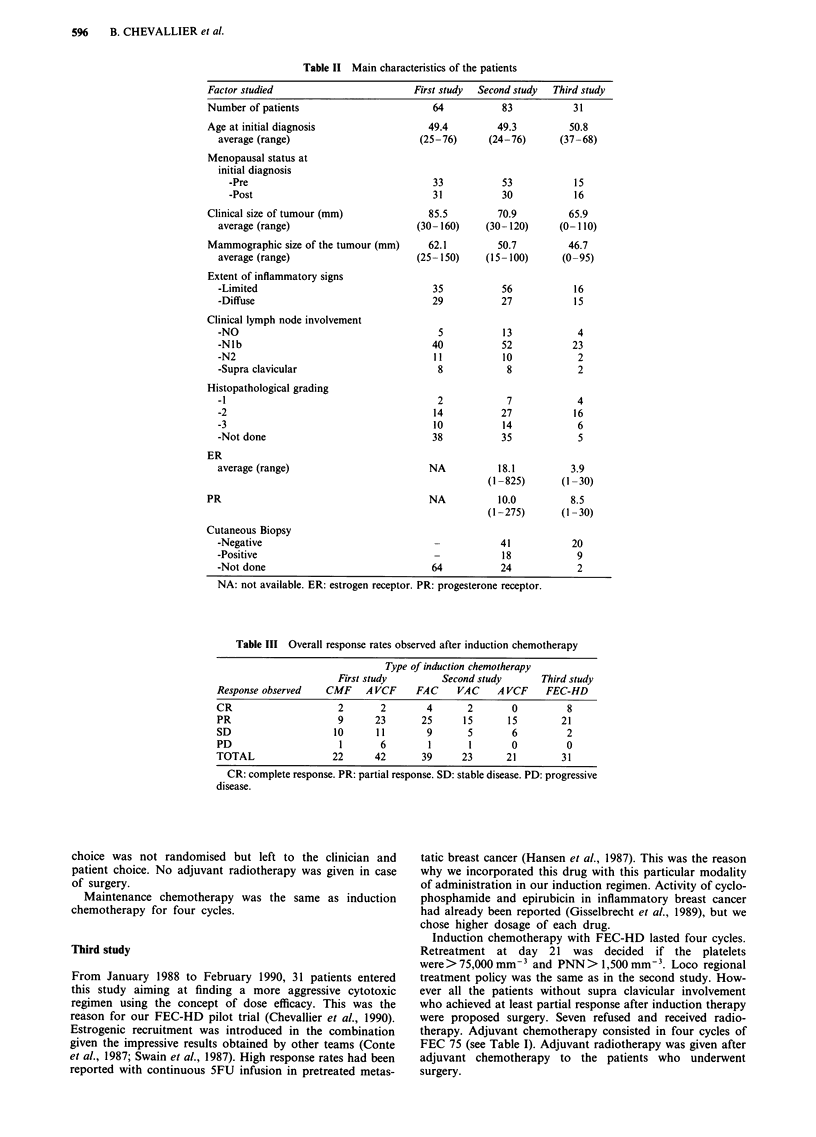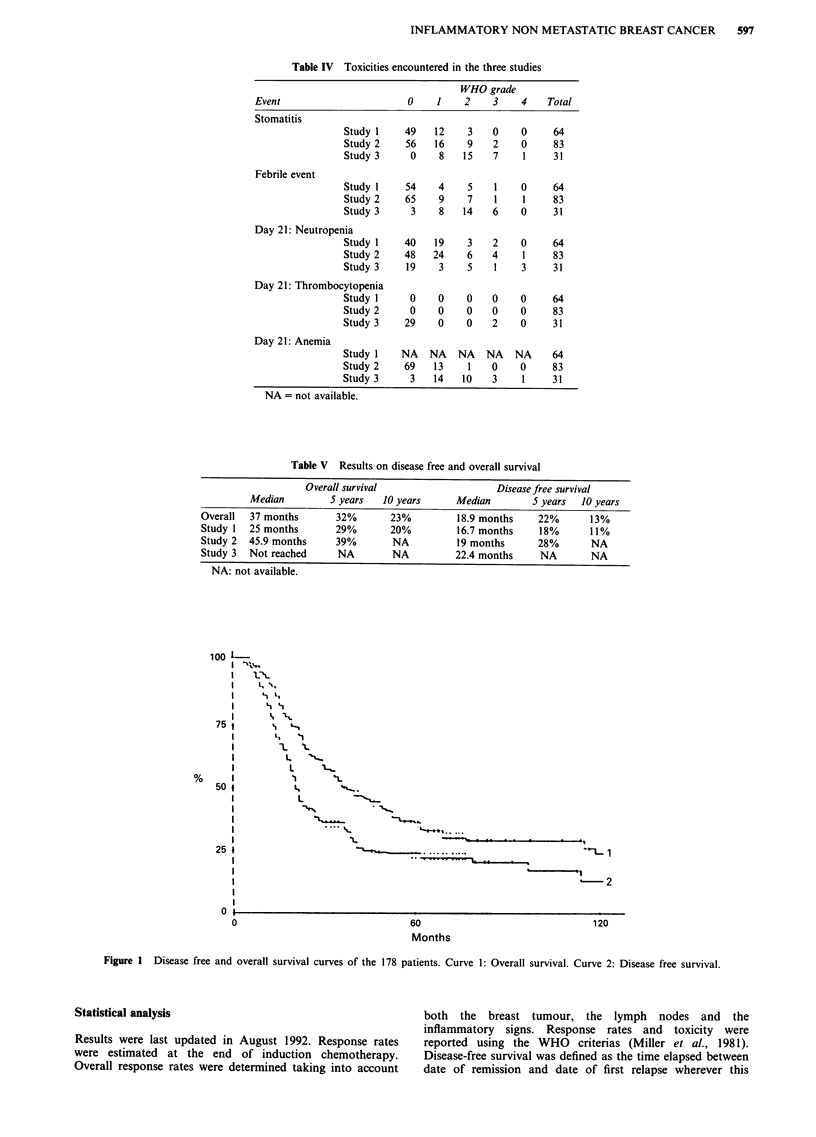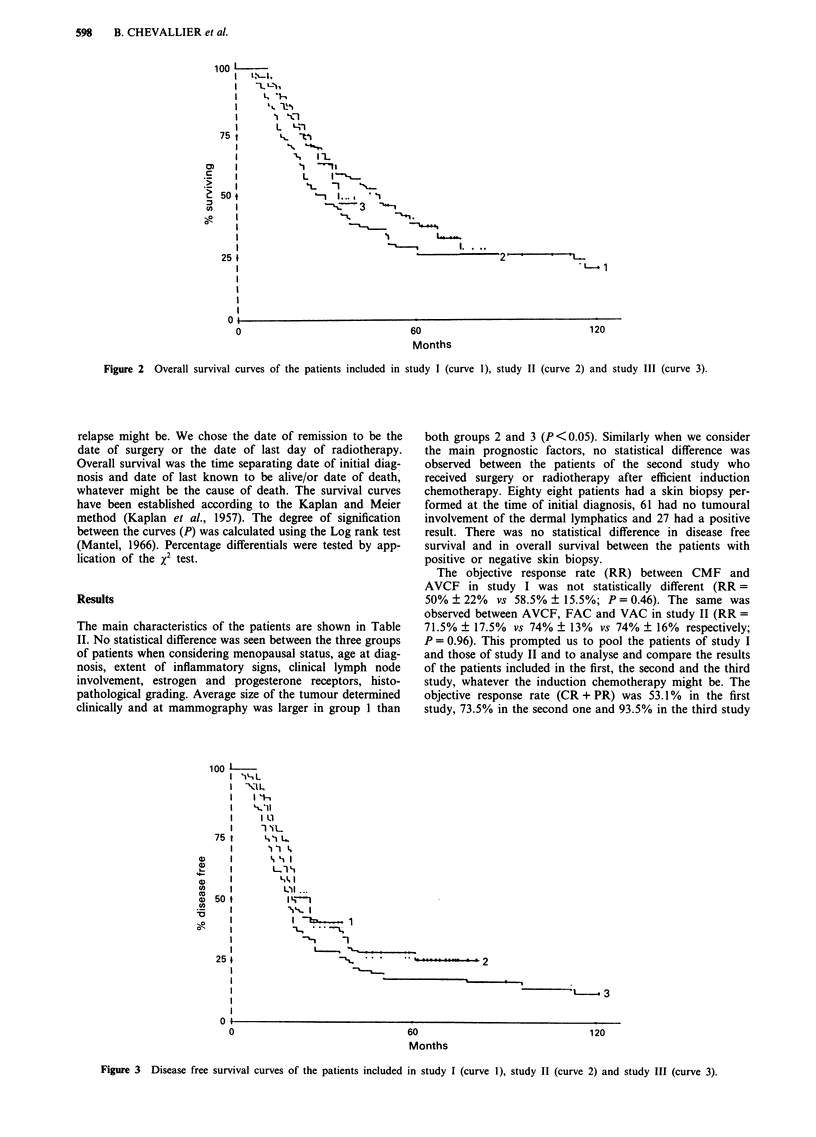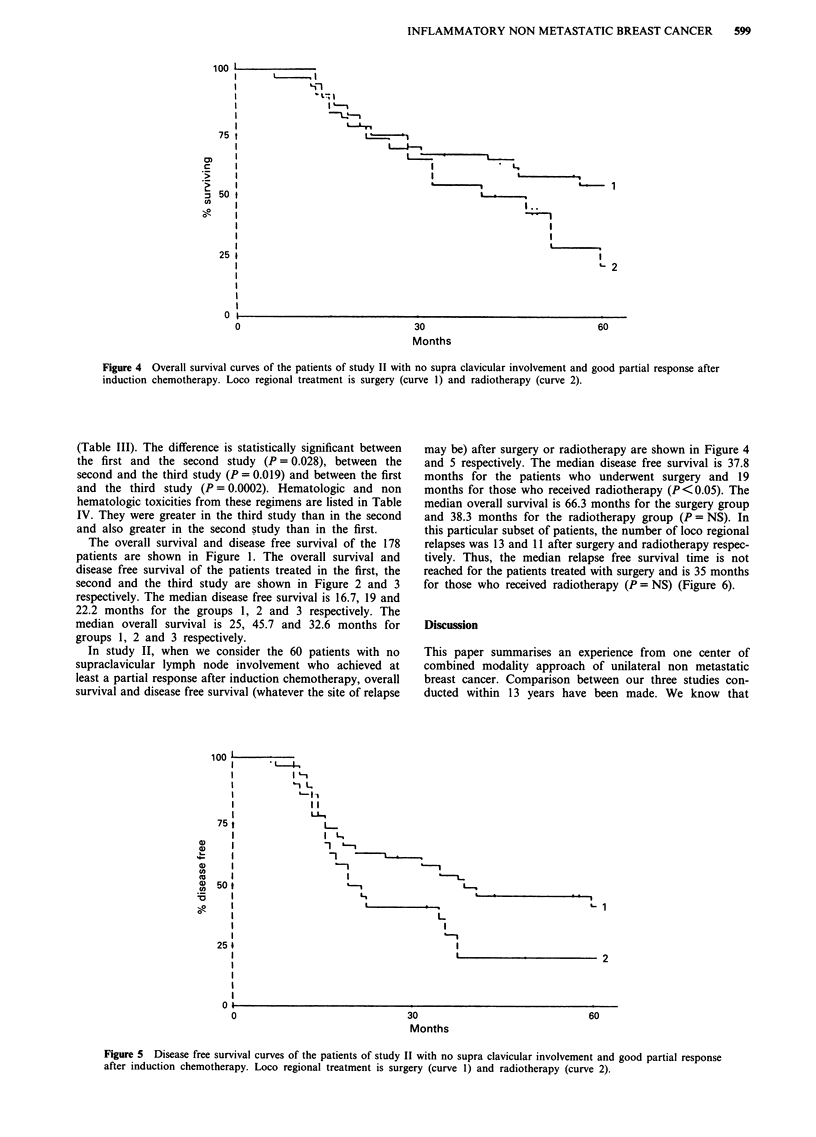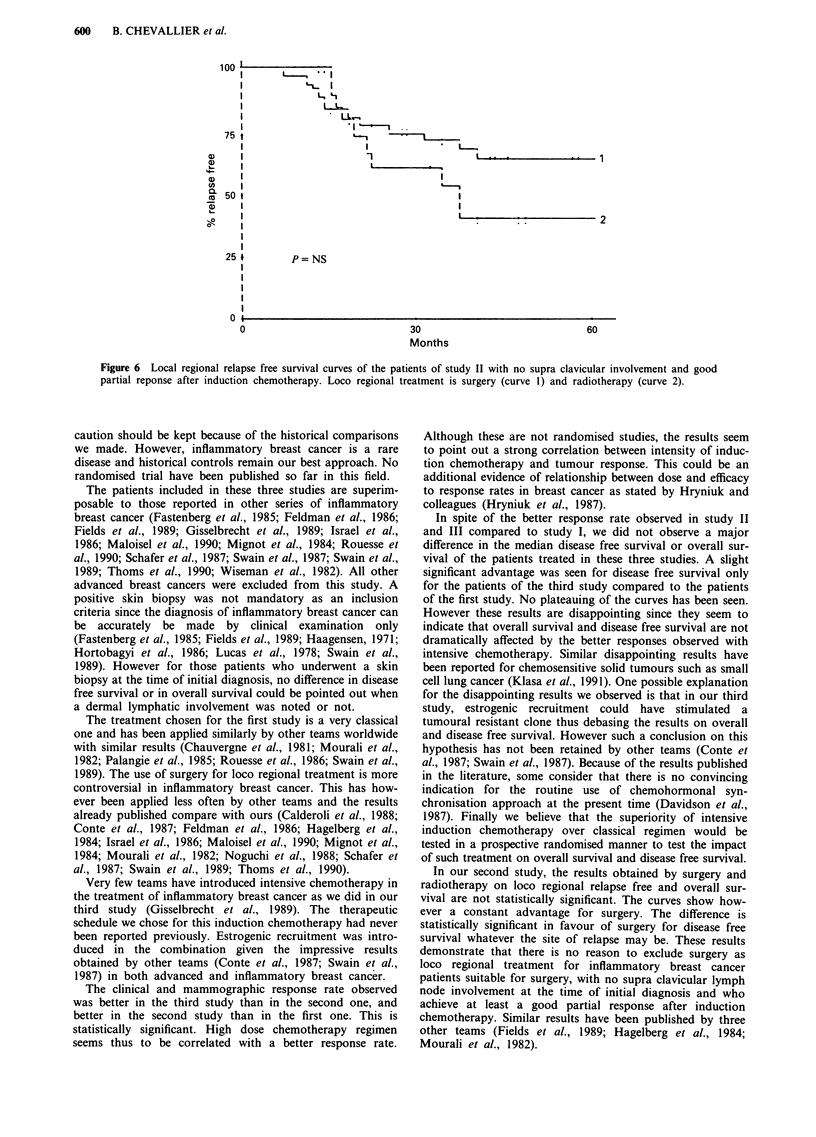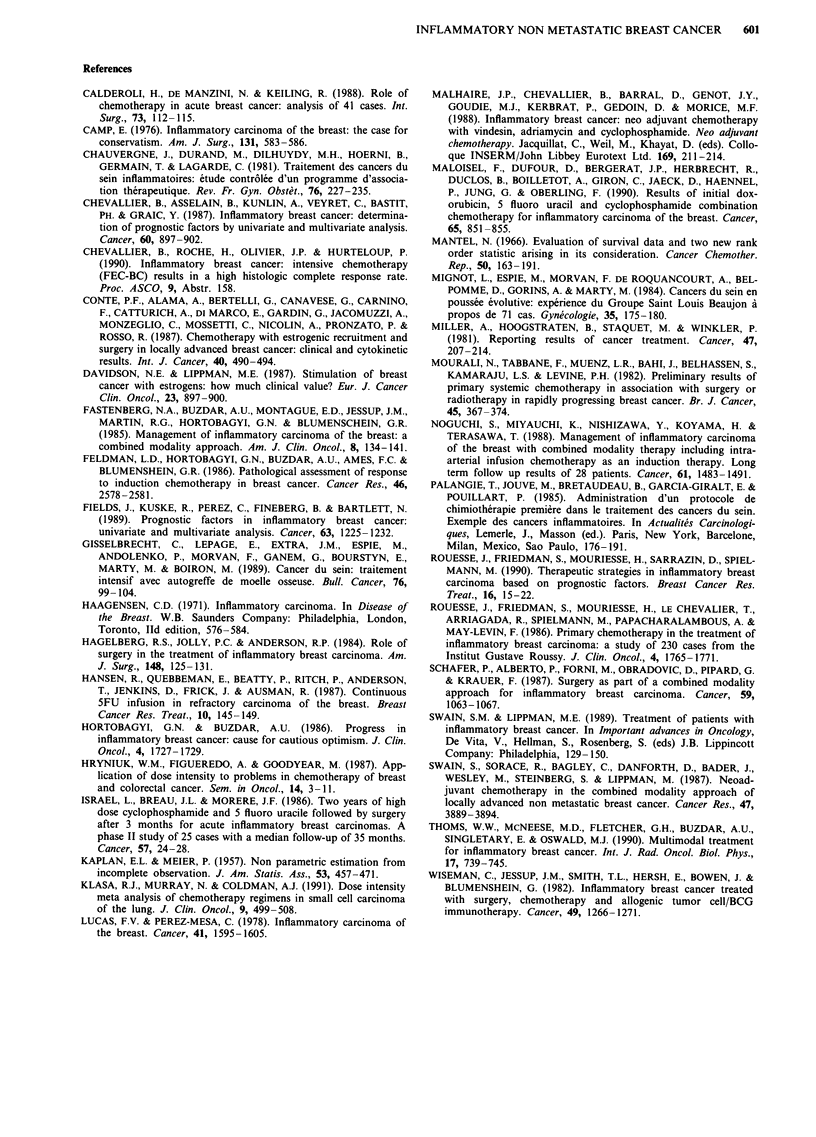# The Centre H. Becquerel studies in inflammatory non metastatic breast cancer. Combined modality approach in 178 patients.

**DOI:** 10.1038/bjc.1993.109

**Published:** 1993-03

**Authors:** B. Chevallier, P. Bastit, Y. Graic, J. F. Menard, J. P. Dauce, J. P. Julien, B. Clavier, A. Kunlin, J. D'Anjou

**Affiliations:** Service d'Oncologie Médicale, Centre H. Becquerel, Rouen, France.

## Abstract

One hundred and seventy-eight patients with non metastatic inflammatory breast cancer (IBC) have been treated at the Centre H. Becquerel. Median follow up is 67 months (6-178). Every patient received neoadjuvant chemotherapy (mean number of cycles = 4; range: 2-8), followed by a loco regional treatment (radiotherapy = XRT or modified radical mastectomy = S), followed by adjuvant chemotherapy. During this period, the types of chemotherapy and locoregional treatment have been the following: Study I: 64 patients treated with CMF or AVCF and XRT; Study II: 83 patients, treated with either AVCF, FAC or VAC followed by S (n = 38) or XRT (n = 22) in case of complete or partial response, or followed by XRT (23) in case of initial supraclavicular lymph node involvement or lack of response after chemotherapy; Study III: 31 patients treated with FEC-HD + Estrogenic recruitment followed by S and XRT after adjuvant chemotherapy, except seven patients who received XRT (refusal of surgery). Although objective response rates (= 56.2, 73.5 and 93.5% for study I, II and III respectively) are statistically better in the 3rd study, this does not translate in dramatically different disease free survival (median = 16.7, 19 and 22.2 months respectively for study I, II and III) or overall survival (median = 25, 45.7 and 32.6 months respectively for study I, II and III). Analysis of subset of patients without supra clavicular lymph node involvement where neoadjuvant chemotherapy obtained at least a 50% response reveals a median disease free survival and median overall survival of respectively 38.3 and 60.1 months for patients who underwent S vs 19 and 38.3 months for those who received XRT (P = 0.15). These studies suggest that surgery has no deleterious effect on outcome of IBC. Advantage on disease free survival or overall survival from intensive chemotherapy in IBC remains to be proven with appropriate randomised trials.


Br. J. Cancer (1993), 67, 594-601 © Macmillan Press Ltd., 1993

### The Centre H. Becquerel studies in inflammatory non metastatic breast cancer. Combined modality approach in 178 patients

B. Chevallier[1], P. Bastit[1], Y. Graic[2], J.F. Menard[3], J.P. Dauce[4], J.P. Julien[4], B. Clavier[4], A. Kunlin[4] & J. D'Anjou[5]

[1]*Service d'Oncologie Médicale, Centre H. Becquerel, 76038, Rouen;* [2]*Service de Radiothérapie, Centre H. Becquerel, 76038, Rouen;* [3]*Service de Biostatistiques, Centre Hospitalier Universitaire, 76038, Rouen;* [4]*Service de Chirurgie, Centre H. Becquerel, 76038, Rouen;* [5]*Service d'Anatomie Pathologique, Centre H. Becquerel, 76038, Rouen, France.*

**Summary** One hundred and seventy-eight patients with non metastatic inflammatory breast cancer (IBC) have been treated at the Centre H. Becquerel. Median follow up is 67 months (6-178). Every patient received neoadjuvant chemotherapy (mean number of cycles = 4; range: 2–8), followed by a loco regional treatment (radiotherapy = XRT or modified radical mastectomy = S), followed by adjuvant chemotherapy. During this period, the types of chemotherapy and locoregional treatment have been the following: Study I: 64 patients treated with CMF or AVCF and XRT; Study II: 83 patients, treated with either AVCF, FAC or VAC followed by S ($n$ = 38) or XRT ($n$ = 22) in case of complete or partial response, or followed by XRT (23) in case of initial supraclavicular lymph node involvement or lack of response after chemotherapy; Study III: 31 patients treated with FEC-HD + Estrogenic recruitment followed by S and XRT after adjuvant chemotherapy, except seven patients who received XRT (refusal of surgery). Although objective response rates ( = 56.2, 73.5 and 93.5% for study I, II and III respectively) are statistically better in the 3rd study, this does not translate in dramatically different disease free survival (median = 16.7, 19 and 22.2 months respectively for study I, II and III) or overall survival (median = 25, 45.7 and 32.6 months respectively for study I, II and III). Analysis of subset of patients without supra clavicular lymph node involvement where neoadjuvant chemotherapy obtained at least a 50% response reveals a median disease free survivcal and median overall survival of respectively 38.3 and 60.1 months for patients who underwent S *vs* 19 and 38.3 months for those who received XRT ($P$ = 0.15). These studies suggest that surgery has no deleterious effect on outcome of IBC. Advantage on disease free survival or overall survival from intensive chemotherapy in IBC remains to be proven with appropriate randomised trials.

Inflammatory adenocarcinoma of the breast is a rare disease with severe prognostic implications due to an almost constant metastatic evolution. The definition is mainly clinical (Haagensen, 1971): enlargement, tenderness, firmness, redness of the breast with usually no fever. A tumour may be clinically detectable or not in the breast. With either surgery or radiotherapy alone or the use of combination surgery and radiotherapy, less than 15% of patients will survive at 5 years (Camp, 1976; Swain, 1989). Because of the high risk of metastasis, a combined modality approach including chemotherapy is at the present time admitted worldwide (Hortobagyi *et al.*, 1986; Swain *et al.*, 1989). This strategy has led to a significant improvement in survival, with 30 to 50% of the patients surviving beyond 5 years (Swain *et al.*, 1989). Achievement of a mastectomy specimen free of residual macroscopic tumour after induction chemotherapy has been shown to be an excellent indicator of a prolonged disease free and overall survival (Feldman *et al.*, 1986). Unfortunately, this situation accounts for less than 20% of the patients (Fastenberg *et al.*, 1985; Israel *et al.*, 1986; Maloisel *et al.*, 1990; Mignot *et al.*, 1984; Schafer *et al.*, 1987; Swain, 1987; Swain *et al.*, 1989; Thomas *et al.*, 1990; Wiseman *et al.*, 1982). One area of research is to increase survival by the search of more efficient induction chemotherapy regimens yielding a higher histologic response rate. More aggressive cytotoxic regimens using the concept of dose efficacy can be tested for this goal. Moreover, the best loco regional treatment is not determined with certainty at the present time for patients who achieve a good objective response to neoadjuvant chemotherapy. For those patients, exclusive radiotherapy remains the academic choice whereas surgery is controversial.

We present here three consecutive studies conducted at the H. Becquerel Cancer Center in 178 patients. The aim of these studies was (1) to improve the response rate obtained with neoadjuvant chemotherapy, (2) to obtain the best locoregional control of the disease, (3) to increase overall survival and disease free survival of these patients with these combined modality approaches.

### Patients and methods

The 178 patients retained for this analysis represent 4% of the newly diagnosed invasive non metastatic adenocarcinomas of the breast between January 1977 and February 1990 ($n$ = 4,359). All of them meet the following criterias: histologically proven invasive adenocarcinoma of one breast, inflammatory signs (erythema + 'peau d'orange + increase of the local heat) involving at least one third of the breast (T4d of the 1988 UICC classification), no history of previous malignancy, no prior specific treatment, serum bilirubin $\leqslant 35\ \mu mol\ l^{-1}$, serum creatinine $\leqslant 130\ \mu mol\ l^{-1}$. A positive skin biopsy was not mandatory for the diagnosis since we considered as others (Fastenberg *et al.*, 1985; Fields *et al.*, 1989; Hortobagyi *et al.*, 1986; Lucas *et al.*, 1978; Swain *et al.*, 1989) that inflammatory breast cancer is a clinical diagnosis rather than a pathologic definition. Only some patients included in the second study and all the patients of the third study had a skin biopsy performed at the time of initial diagnosis ($n$ = 88). Cases with previous history of breast cancer, bilateral breast cancer, prior history of heart failure or with an history of another malignant tumour were not kept for this analysis. This excludes also advanced breast cancer such as scirrous or ulcerated cancers and also rapidly progressing breast cancers without inflammatory signs. Since January 1977, every patient had a pretreatment check-up including history and physical examination, bilateral mammographies, chest X ray, bone scan, liver echography or CT scan, EKG and echocardiography or radionuclide cardiac scan (since 1983), complete blood count, standard biological tests, carcinoembryonic antigen (until 1986), CA 15.3 (since 1987). Estrogen and progesterone receptors were measured accord-

Correspondence: B. Chevallier, Centre H. Becquerel, Rue d'Amiens, 76038 Rouen Cédex, France.
Received 4 March 1992; and in revised form 4 September 1992.

ing to the coal-dextran technique for the patients of the second and third study. Improvement in X-ray and echographic techniques and the use of more sensitive tumour markers could have resulted in a more accurate staging for the more recent patients.

Assessment of tumour response was performed at least every two cycles with clinical examination and mammographies. All baseline investigations were repeated at the end of the treatment.

Follow-up was every 3 months the first year, bi-annual for the next 4 years and once a year therafter, with clinical examination, chest X ray and biochemical screen. Mammographies were performed once a year. Locoregional or controlateral relapses were confirmed by biopsies or unequivocal radiological evidence.

### Treatment modalities

The patients of each of the three groups were treated with a combined modality approach. Induction chemotherapy was always given first for an average of four cycles (range: 2–8). Loco regional treatment consisted of radiotherapy or surgery. Radiotherapy as exclusive loco regional treatment employed Cobalt 60 and delivered 60 grays to the tumour, the whole breast and the axilla, 50 grays to the internal mammary chain and 46 grays to the supraclavicular area. Ten Grays per week were given in five fractions. Four to 6 weeks after the first irradiation, a 20 grays boost was given to the breast tumoural remainders. No chemotherapy was administered during radiotherapy. Surgery consisted in every case of a modified radical mastectomy with homolateral axillary clearance. No conservative surgery was performed. Maintenance chemotherapy was administered only if induction chemotherapy obtained at least a partial response and used the same schedule as induction chemotherapy for an average of four cycles (range: 0–12). No standard protocol was adopted in the treatment of loco regional or distant recurrences: patients were given the most suitable therapy for their state.

Between 1977 and 1990 modifications in the treatment programme have been made in three steps.

### First study

In January 1977, induction chemotherapy was introduced at the Centre H. Becquerel before locoregional treatment. This induction chemotherapy consisted either in CMF ($n = 22$; until April 1979) or AVCF ($n = 42$) (see Table I) given for three cycles. Retreatment was decided at day 28 if platelets were $> 100000\ mm^{-3}$ and PNN $> 2000\ mm^{-3}$. Loco regional treatment consisted in every case in exclusive radiotherapy. Surgery was only employed as a salvage treatment in case of loco regional relapse after radiotherapy ($n = 8$). Maintenance chemotherapy was the same as induction chemotherapy for eight cycles. From January 1977 to June 1983 64 patients have been included in this study (Chevallier *et al.*, 1987).

### Second study

From July 1983 to December 1987, a total of 83 patients have been included in this second study. Because of the poor results in loco regional control with high incidence of intramammary remainders and loco regional relapses observed in the first study (Chevallier *et al.*, 1987), we decided to test the value of surgery as loco regional treatment. This was done for the patients with no supra clavicular lymph node involvement achieving at least a good partial response with disappearance of erythema after induction chemotherapy. This chemotherapy consisted either of AVCF ($n = 21$) FAC ($n = 39$) or VAC ($n = 23$) (see Table I). Two to six cycles were given (average = four cycles). One patient received eight cycles. Policy for retreatment at day 28 for the patients treated with AVCF was the same as in the first study. The same blood count criteria were used for the patients treated with FAC or VAC but retreatment was planned to be at day 21 instead of day 28.

Twenty-three patients had either supra clavicular lymph node involvement or achieved stabilisation or progressive disease after induction therapy. These 23 patients received exclusive radiotherapy for loco regional treatment.

Sixty patients without supra clavicular lymph node involvement and who achieved at least a partial response after neoadjuvant chemotherapy were either operated on ($n = 38$) or received exclusive radiotherapy ($n = 22$). This

**Table I** Chemotherapy schedules employed in the three studies

| *Chemotherapy regimen* | *Days* | *Drugs and dosage* | *Number of patients* |
|---|---|---|---|
| CMF | 1 and 8 | 5 Fluoro Uracil 600 mg/m²* | |
| | 1 and 8 | Methotrexate 40 mg/m²* | 22 |
| | 1 to 7 | Cyclophosphamide 150 mg/m²† | |
| AVCF | 1 | Doxorubicin 30 mg/m² | |
| | 2 | Vincristin 1.4 mg/m² (max = 2 mg)* | 63 |
| | 3 to 6 | Cyclophosphamide 300 mg/m²* and | |
| | 3 to 6 | 5 fluoro uracil 400 mg/m²* | |
| VAC | 1 to 4 | Vindesin 1 mg/m²* | |
| | 1 to 3 | Doxorubicin 15 mg/m²* | 23 |
| | 1 to 3 | Cyclophosphamide 200 mg/m² | |
| FAC | 1 | Doxorubicin 50 mg/m²* | |
| | 1 | 5 Fluoro uracil 500 mg/m²* | 39 |
| | 1 | Cyclophosphamide 500 ng m²* | |
| FEC-HD | 1 and 2 | Ethinyl oestradiol 50 μg TID† | |
| | 2 to 5 | 5 Fluoro uracil 750 mg/m² (CIVI) | 31 |
| | 3 to 5 | Epirubicin 35 mg/m²* | |
| | 3 to 5 | Cyclophosphamide 400 mg/m²* | |
| FEC 75 | 1 | Epirubicin 75 mg/m²* | |
| (Adjuvant) | 1 | 5 Fluoro uracil 500 mg/m²* | 31 |
| | 1 | Cyclophosphamide 500 mg/m²* | |
| | | TOTAL = | 178 |

CIVI: continuous i.v. infusion. *: bolus i.v. infusion. †: given orally.

**Table II** Main characteristics of the patients

| Factor studied | First study | Second study | Third study |
|---|---|---|---|
| Number of patients | 64 | 83 | 31 |
| Age at initial diagnosis | 49.4 | 49.3 | 50.8 |
| average (range) | (25-76) | (24-76) | (37-68) |
| Menopausal status at initial diagnosis | | | |
| -Pre | 33 | 53 | 15 |
| -Post | 31 | 30 | 16 |
| Clinical size of tumour (mm) | 85.5 | 70.9 | 65.9 |
| average (range) | (30-160) | (30-120) | (0-110) |
| Mammographic size of the tumour (mm) | 62.1 | 50.7 | 46.7 |
| average (range) | (25-150) | (15-100) | (0-95) |
| Extent of inflammatory signs | | | |
| -Limited | 35 | 56 | 16 |
| -Diffuse | 29 | 27 | 15 |
| Clinical lymph node involvement | | | |
| -NO | 5 | 13 | 4 |
| -N1b | 40 | 52 | 23 |
| -N2 | 11 | 10 | 2 |
| -Supra clavicular | 8 | 8 | 2 |
| Histopathological grading | | | |
| -1 | 2 | 7 | 4 |
| -2 | 14 | 27 | 16 |
| -3 | 10 | 14 | 6 |
| -Not done | 38 | 35 | 5 |
| ER | | | |
| average (range) | NA | 18.1 (1-825) | 3.9 (1-30) |
| PR | NA | 10.0 (1-275) | 8.5 (1-30) |
| Cutaneous Biopsy | | | |
| -Negative | – | 41 | 20 |
| -Positive | – | 18 | 9 |
| -Not done | 64 | 24 | 2 |

NA: not available. ER: estrogen receptor. PR: progesterone receptor.

**Table III** Overall response rates observed after induction chemotherapy

| | Type of induction chemotherapy | | | | | |
|---|---|---|---|---|---|---|
| | First study | | Second study | | | Third study |
| Response observed | CMF | AVCF | FAC | VAC | AVCF | FEC-HD |
| CR | 2 | 2 | 4 | 2 | 0 | 8 |
| PR | 9 | 23 | 25 | 15 | 15 | 21 |
| SD | 10 | 11 | 9 | 5 | 6 | 2 |
| PD | 1 | 6 | 1 | 1 | 0 | 0 |
| TOTAL | 22 | 42 | 39 | 23 | 21 | 31 |

CR: complete response. PR: partial response. SD: stable disease. PD: progressive disease.

choice was not randomised but left to the clinician and patient choice. No adjuvant radiotherapy was given in case of surgery.

Maintenance chemotherapy was the same as induction chemotherapy for four cycles.

### Third study

From January 1988 to February 1990, 31 patients entered this study aiming at finding a more aggressive cytotoxic regimen using the concept of dose efficacy. This was the reason for our FEC-HD pilot trial (Chevallier et al., 1990). Estrogenic recruitment was introduced in the combination given the impressive results obtained by other teams (Conte et al., 1987; Swain et al., 1987). High response rates had been reported with continuous 5FU infusion in pretreated metastatic breast cancer (Hansen et al., 1987). This was the reason why we incorporated this drug with this particular modality of administration in our induction regimen. Activity of cyclophosphamide and epirubicin in inflammatory breast cancer had already been reported (Gisselbrecht et al., 1989), but we chose higher dosage of each drug.

Induction chemotherapy with FEC-HD lasted four cycles. Retreatment at day 21 was decided if the platelets were $> 75,000\ mm^{-3}$ and PNN $> 1,500\ mm^{-3}$. Loco regional treatment policy was the same as in the second study. However all the patients without supra clavicular involvement who achieved at least partial response after induction therapy were proposed surgery. Seven refused and received radiotherapy. Adjuvant chemotherapy consisted in four cycles of FEC 75 (see Table I). Adjuvant radiotherapy was given after adjuvant chemotherapy to the patients who underwent surgery.

**Table IV** Toxicities encountered in the three studies

| Event | | WHO grade 0 | 1 | 2 | 3 | 4 | Total |
|---|---|---|---|---|---|---|---|
| Stomatitis | | | | | | | |
| | Study 1 | 49 | 12 | 3 | 0 | 0 | 64 |
| | Study 2 | 56 | 16 | 9 | 2 | 0 | 83 |
| | Study 3 | 0 | 8 | 15 | 7 | 1 | 31 |
| Febrile event | | | | | | | |
| | Study 1 | 54 | 4 | 5 | 1 | 0 | 64 |
| | Study 2 | 65 | 9 | 7 | 1 | 1 | 83 |
| | Study 3 | 3 | 8 | 14 | 6 | 0 | 31 |
| Day 21: Neutropenia | | | | | | | |
| | Study 1 | 40 | 19 | 3 | 2 | 0 | 64 |
| | Study 2 | 48 | 24 | 6 | 4 | 1 | 83 |
| | Study 3 | 19 | 3 | 5 | 1 | 3 | 31 |
| Day 21: Thrombocytopenia | | | | | | | |
| | Study 1 | 0 | 0 | 0 | 0 | 0 | 64 |
| | Study 2 | 0 | 0 | 0 | 0 | 0 | 83 |
| | Study 3 | 29 | 0 | 0 | 2 | 0 | 31 |
| Day 21: Anemia | | | | | | | |
| | Study 1 | NA | NA | NA | NA | NA | 64 |
| | Study 2 | 69 | 13 | 1 | 0 | 0 | 83 |
| | Study 3 | 3 | 14 | 10 | 3 | 1 | 31 |

NA = not available.

**Table V** Results on disease free and overall survival

| | Overall survival Median | 5 years | 10 years | Disease free survival Median | 5 years | 10 years |
|---|---|---|---|---|---|---|
| Overall | 37 months | 32% | 23% | 18.9 months | 22% | 13% |
| Study 1 | 25 months | 29% | 20% | 16.7 months | 18% | 11% |
| Study 2 | 45.9 months | 39% | NA | 19 months | 28% | NA |
| Study 3 | Not reached | NA | NA | 22.4 months | NA | NA |

NA: not available.

**Figure 1** Disease free and overall survival curves of the 178 patients. Curve 1: Overall survival. Curve 2: Disease free survival.

### Statistical analysis

Results were last updated in August 1992. Response rates were estimated at the end of induction chemotherapy. Overall response rates were determined taking into account both the breast tumour, the lymph nodes and the inflammatory signs. Response rates and toxicity were reported using the WHO criterias (Miller *et al.*, 1981). Disease-free survival was defined as the time elapsed between date of remission and date of first relapse wherever this

**Figure 2** Overall survival curves of the patients included in study I (curve 1), study II (curve 2) and study III (curve 3).

relapse might be. We chose the date of remission to be the date of surgery or the date of last day of radiotherapy. Overall survival was the time separating date of initial diagnosis and date of last known to be alive/or date of death, whatever might be the cause of death. The survival curves have been established according to the Kaplan and Meier method (Kaplan *et al.*, 1957). The degree of signification between the curves ($P$) was calculated using the Log rank test (Mantel, 1966). Percentage differentials were tested by application of the $\chi^2$ test.

### Results

The main characteristics of the patients are shown in Table II. No statistical difference was seen between the three groups of patients when considering menopausal status, age at diagnosis, extent of inflammatory signs, clinical lymph node involvement, estrogen and progesterone receptors, histopathological grading. Average size of the tumour determined clinically and at mammography was larger in group 1 than both groups 2 and 3 ($P<0.05$). Similarly when we consider the main prognostic factors, no statistical difference was observed between the patients of the second study who received surgery or radiotherapy after efficient induction chemotherapy. Eighty eight patients had a skin biopsy performed at the time of initial diagnosis, 61 had no tumoural involvement of the dermal lymphatics and 27 had a positive result. There was no statistical difference in disease free survival and in overall survival between the patients with positive or negative skin biopsy.

The objective response rate (RR) between CMF and AVCF in study I was not statistically different (RR = 50% ± 22% *vs* 58.5% ± 15.5%; $P=0.46$). The same was observed between AVCF, FAC and VAC in study II (RR = 71.5% ± 17.5% *vs* 74% ± 13% *vs* 74% ± 16% respectively; $P=0.96$). This prompted us to pool the patients of study I and those of study II and to analyse and compare the results of the patients included in the first, the second and the third study, whatever the induction chemotherapy might be. The objective response rate (CR + PR) was 53.1% in the first study, 73.5% in the second one and 93.5% in the third study

**Figure 3** Disease free survival curves of the patients included in study I (curve 1), study II (curve 2) and study III (curve 3).

**Figure 4** Overall survival curves of the patients of study II with no supra clavicular involvement and good partial response after induction chemotherapy. Loco regional treatment is surgery (curve 1) and radiotherapy (curve 2).

(Table III). The difference is statistically significant between the first and the second study ($P = 0.028$), between the second and the third study ($P = 0.019$) and between the first and the third study ($P = 0.0002$). Hematologic and non hematologic toxicities from these regimens are listed in Table IV. They were greater in the third study than in the second and also greater in the second study than in the first.

The overall survival and disease free survival of the 178 patients are shown in Figure 1. The overall survival and disease free survival of the patients treated in the first, the second and the third study are shown in Figure 2 and 3 respectively. The median disease free survival is 16.7, 19 and 22.2 months for the groups 1, 2 and 3 respectively. The median overall survival is 25, 45.7 and 32.6 months for groups 1, 2 and 3 respectively.

In study II, when we consider the 60 patients with no supraclavicular lymph node involvement who achieved at least a partial response after induction chemotherapy, overall survival and disease free survival (whatever the site of relapse may be) after surgery or radiotherapy are shown in Figure 4 and 5 respectively. The median disease free survival is 37.8 months for the patients who underwent surgery and 19 months for those who received radiotherapy ($P < 0.05$). The median overall survival is 66.3 months for the surgery group and 38.3 months for the radiotherapy group ($P$ = NS). In this particular subset of patients, the number of loco regional relapses was 13 and 11 after surgery and radiotherapy respectively. Thus, the median relapse free survival time is not reached for the patients treated with surgery and is 35 months for those who received radiotherapy ($P$ = NS) (Figure 6).

### Discussion

This paper summarises an experience from one center of combined modality approach of unilateral non metastatic breast cancer. Comparison between our three studies conducted within 13 years have been made. We know that

**Figure 5** Disease free survival curves of the patients of study II with no supra clavicular involvement and good partial response after induction chemotherapy. Loco regional treatment is surgery (curve 1) and radiotherapy (curve 2).

**Figure 6** Local regional relapse free survival curves of the patients of study II with no supra clavicular involvement and good partial reponse after induction chemotherapy. Loco regional treatment is surgery (curve 1) and radiotherapy (curve 2).

caution should be kept because of the historical comparisons we made. However, inflammatory breast cancer is a rare disease and historical controls remain our best approach. No randomised trial have been published so far in this field.

The patients included in these three studies are superimposable to those reported in other series of inflammatory breast cancer (Fastenberg *et al.*, 1985; Feldman *et al.*, 1986; Fields *et al.*, 1989; Gisselbrecht *et al.*, 1989; Israel *et al.*, 1986; Maloisel *et al.*, 1990; Mignot *et al.*, 1984; Rouesse *et al.*, 1990; Schafer *et al.*, 1987; Swain *et al.*, 1987; Swain *et al.*, 1989; Thoms *et al.*, 1990; Wiseman *et al.*, 1982). All other advanced breast cancers were excluded from this study. A positive skin biopsy was not mandatory as an inclusion criteria since the diagnosis of inflammatory breast cancer can be accurately be made by clinical examination only (Fastenberg *et al.*, 1985; Fields *et al.*, 1989; Haagensen, 1971; Hortobagyi *et al.*, 1986; Lucas *et al.*, 1978; Swain *et al.*, 1989). However for those patients who underwent a skin biopsy at the time of initial diagnosis, no difference in disease free survival or in overall survival could be pointed out when a dermal lymphatic involvement was noted or not.

The treatment chosen for the first study is a very classical one and has been applied similarly by other teams worldwide with similar results (Chauvergne *et al.*, 1981; Mourali *et al.*, 1982; Palangie *et al.*, 1985; Rouesse *et al.*, 1986; Swain *et al.*, 1989). The use of surgery for loco regional treatment is more controversial in inflammatory breast cancer. This has however been applied less often by other teams and the results already published compare with ours (Calderoli *et al.*, 1988; Conte *et al.*, 1987; Feldman *et al.*, 1986; Hagelberg *et al.*, 1984; Israel *et al.*, 1986; Maloisel *et al.*, 1990; Mignot *et al.*, 1984; Mourali *et al.*, 1982; Noguchi *et al.*, 1988; Schafer *et al.*, 1987; Swain *et al.*, 1989; Thoms *et al.*, 1990).

Very few teams have introduced intensive chemotherapy in the treatment of inflammatory breast cancer as we did in our third study (Gisselbrecht *et al.*, 1989). The therapeutic schedule we chose for this induction chemotherapy had never been reported previously. Estrogenic recruitment was introduced in the combination given the impressive results obtained by other teams (Conte *et al.*, 1987; Swain *et al.*, 1987) in both advanced and inflammatory breast cancer.

The clinical and mammographic response rate observed was better in the third study than in the second one, and better in the second study than in the first one. This is statistically significant. High dose chemotherapy regimen seems thus to be correlated with a better response rate. Although these are not randomised studies, the results seem to point out a strong correlation between intensity of induction chemotherapy and tumour response. This could be an additional evidence of relationship between dose and efficacy to response rates in breast cancer as stated by Hryniuk and colleagues (Hryniuk *et al.*, 1987).

In spite of the better response rate observed in study II and III compared to study I, we did not observe a major difference in the median disease free survival or overall survival of the patients treated in these three studies. A slight significant advantage was seen for disease free survival only for the patients of the third study compared to the patients of the first study. No plateauing of the curves has been seen. However these results are disappointing since they seem to indicate that overall survival and disease free survival are not dramatically affected by the better responses observed with intensive chemotherapy. Similar disappointing results have been reported for chemosensitive solid tumours such as small cell lung cancer (Klasa *et al.*, 1991). One possible explanation for the disappointing results we observed is that in our third study, estrogenic recruitment could have stimulated a tumoural resistant clone thus debasing the results on overall and disease free survival. However such a conclusion on this hypothesis has not been retained by other teams (Conte *et al.*, 1987; Swain *et al.*, 1987). Because of the results published in the literature, some consider that there is no convincing indication for the routine use of chemohormonal synchronisation approach at the present time (Davidson *et al.*, 1987). Finally we believe that the superiority of intensive induction chemotherapy over classical regimen would be tested in a prospective randomised manner to test the impact of such treatment on overall survival and disease free survival.

In our second study, the results obtained by surgery and radiotherapy on loco regional relapse free and overall survival are not statistically significant. The curves show however a constant advantage for surgery. The difference is statistically significant in favour of surgery for disease free survival whatever the site of relapse may be. These results demonstrate that there is no reason to exclude surgery as loco regional treatment for inflammatory breast cancer patients suitable for surgery, with no supra clavicular lymph node involvement at the time of initial diagnosis and who achieve at least a good partial response after induction chemotherapy. Similar results have been published by three other teams (Fields *et al.*, 1989; Hagelberg *et al.*, 1984; Mourali *et al.*, 1982).

### References

CALDEROLI, H., DE MANZINI, N. & KEILING, R. (1988). Role of chemotherapy in acute breast cancer: analysis of 41 cases. *Int. Surg.*, **73**, 112–115.

CAMP, E. (1976). Inflammatory carcinoma of the breast: the case for conservatism. *Am. J. Surg.*, **131**, 583–586.

CHAUVERGNE, J., DURAND, M., DILHUYDY, M.H., HOERNI, B., GERMAIN, T. & LAGARDE, C. (1981). Traitement des cancers du sein inflammatoires: étude contrôlée d'un programme d'association thérapeutique. *Rev. Fr. Gyn. Obstèt.*, **76**, 227–235.

CHEVALLIER, B., ASSELAIN, B., KUNLIN, A., VEYRET, C., BASTIT, PH. & GRAIC, Y. (1987). Inflammatory breast cancer: determination of prognostic factors by univariate and multivariate analysis. *Cancer*, **60**, 897–902.

CHEVALLIER, B., ROCHE, H., OLIVIER, J.P. & HURTELOUP, P. (1990). Inflammatory breast cancer: intensive chemotherapy (FEC-BC) results in a high histologic complete response rate. *Proc. ASCO*, **9**, Abstr. 158.

CONTE, P.F., ALAMA, A., BERTELLI, G., CANAVESE, G., CARNINO, F., CATTURICH, A., DI MARCO, E., GARDIN, G., JACOMUZZI, A., MONZEGLIO, C., MOSSETTI, C., NICOLIN, A., PRONZATO, P. & ROSSO, R. (1987). Chemotherapy with estrogenic recruitment and surgery in locally advanced breast cancer: clinical and cytokinetic results. *Int. J. Cancer*, **40**, 490–494.

DAVIDSON, N.E. & LIPPMAN, M.E. (1987). Stimulation of breast cancer with estrogens: how much clinical value? *Eur. J. Cancer Clin. Oncol.*, **23**, 897–900.

FASTENBERG, N.A., BUZDAR, A.U., MONTAGUE, E.D., JESSUP, J.M., MARTIN, R.G., HORTOBAGYI, G.N. & BLUMENSCHEIN, G.R. (1985). Management of inflammatory carcinoma of the breast: a combined modality approach. *Am. J. Clin. Oncol.*, **8**, 134–141.

FELDMAN, L.D., HORTOBAGYI, G.N., BUZDAR, A.U., AMES, F.C. & BLUMENSHEIN, G.R. (1986). Pathological assessment of response to induction chemotherapy in breast cancer. *Cancer Res.*, **46**, 2578–2581.

FIELDS, J., KUSKE, R., PEREZ, C., FINEBERG, B. & BARTLETT, N. (1989). Prognostic factors in inflammatory breast cancer: univariate and multivariate analysis. *Cancer*, **63**, 1225–1232.

GISSELBRECHT, C., LEPAGE, E., EXTRA, J.M., ESPIE, M., ANDOLENKO, P., MORVAN, F., GANEM, G., BOURSTYN, E., MARTY, M. & BOIRON, M. (1989). Cancer du sein: traitement intensif avec autogreffe de moelle osseuse. *Bull. Cancer*, **76**, 99–104.

HAAGENSEN, C.D. (1971). Inflammatory carcinoma. In *Disease of the Breast*. W.B. Saunders Company: Philadelphia, London, Toronto, IId edition, 576–584.

HAGELBERG, R.S., JOLLY, P.C. & ANDERSON, R.P. (1984). Role of surgery in the treatment of inflammatory breast carcinoma. *Am. J. Surg.*, **148**, 125–131.

HANSEN, R., QUEBBEMAN, E., BEATTY, P., RITCH, P., ANDERSON, T., JENKINS, D., FRICK, J. & AUSMAN, R. (1987). Continuous 5FU infusion in refractory carcinoma of the breast. *Breast Cancer Res. Treat.*, **10**, 145–149.

HORTOBAGYI, G.N. & BUZDAR, A.U (1986). Progress in inflammatory breast cancer: cause for cautious optimism. *J. Clin. Oncol.*, **4**, 1727–1729.

HRYNIUK, W.M., FIGUEREDO, A. & GOODYEAR, M. (1987). Application of dose intensity to problems in chemotherapy of breast and colorectal cancer. *Sem. in Oncol.*, **14**, 3–11.

ISRAEL, L., BREAU, J.L. & MORERE, J.F. (1986). Two years of high dose cyclophosphamide and 5 fluoro uracile followed by surgery after 3 months for acute inflammatory breast carcinomas. A phase II study of 25 cases with a median follow-up of 35 months. *Cancer*, **57**, 24–28.

KAPLAN, E.L. & MEIER, P. (1957). Non parametric estimation from incomplete observation. *J. Am. Statis. Ass.*, **53**, 457–471.

KLASA, R.J., MURRAY, N. & COLDMAN, A.J. (1991). Dose intensity meta analysis of chemotherapy regimens in small cell carcinoma of the lung. *J. Clin. Oncol.*, **9**, 499–508.

LUCAS, F.V. & PEREZ-MESA, C. (1978). Inflammatory carcinoma of the breast. *Cancer*, **41**, 1595–1605.

MALHAIRE, J.P., CHEVALLIER, B., BARRAL, D., GENOT, J.Y., GOUDIE, M.J., KERBRAT, P., GEDOIN, D. & MORICE, M.F. (1988). Inflammatory breast cancer: neo adjuvant chemotherapy with vindesin, adriamycin and cyclophosphamide. *Neo adjuvant chemotherapy*. Jacquillat, C., Weil, M., Khayat, D. (eds). Colloque INSERM/John Libbey Eurotext Ltd. **169**, 211–214.

MALOISEL, F., DUFOUR, D., BERGERAT, J.P., HERBRECHT, R., DUCLOS, B., BOILLETOT, A., GIRON, C., JAECK, D., HAENNEL, P., JUNG, G. & OBERLING, F. (1990). Results of initial doxorubicin, 5 fluoro uracil and cyclophosphamide combination chemotherapy for inflammatory carcinoma of the breast. *Cancer*, **65**, 851–855.

MANTEL, N. (1966). Evaluation of survival data and two new rank order statistic arising in its consideration. *Cancer Chemother. Rep.*, **50**, 163–191.

MIGNOT, L., ESPIE, M., MORVAN, F. DE ROQUANCOURT, A., BELPOMME, D., GORINS, A. & MARTY, M. (1984). Cancers du sein en poussée évolutive: expérience du Groupe Saint Louis Beaujon à propos de 71 cas. *Gynécologie*, **35**, 175–180.

MILLER, A., HOOGSTRATEN, B., STAQUET, M. & WINKLER, P. (1981). Reporting results of cancer treatment. *Cancer*, **47**, 207–214.

MOURALI, N., TABBANE, F., MUENZ, L.R., BAHI, J., BELHASSEN, S., KAMARAJU, L.S. & LEVINE, P.H. (1982). Preliminary results of primary systemic chemotherapy in association with surgery or radiotherapy in rapidly progressing breast cancer. *Br. J. Cancer*, **45**, 367–374.

NOGUCHI, S., MIYAUCHI, K., NISHIZAWA, Y., KOYAMA, H. & TERASAWA, T. (1988). Management of inflammatory carcinoma of the breast with combined modality therapy including intraarterial infusion chemotherapy as an induction therapy. Long term follow up results of 28 patients. *Cancer*, **61**, 1483–1491.

PALANGIE, T., JOUVE, M., BRETAUDEAU, B., GARCIA-GIRALT, E. & POUILLART, P. (1985). Administration d'un protocole de chimiothérapie première dans le traitement des cancers du sein. Exemple des cancers inflammatoires. In *Actualités Carcinologiques*, Lemerle, J., Masson (ed.). Paris, New York, Barcelone, Milan, Mexico, Sao Paulo, 176–191.

ROUESSE, J., FRIEDMAN, S., MOURIESSE, H., SARRAZIN, D., SPIELMANN, M. (1990). Therapeutic strategies in inflammatory breast carcinoma based on prognostic factors. *Breast Cancer Res. Treat.*, **16**, 15–22.

ROUESSE, J., FRIEDMAN, S., MOURIESSE, H., LE CHEVALIER, T., ARRIAGADA, R., SPIELMANN, M., PAPACHARALAMBOUS, A. & MAY-LEVIN, F. (1986). Primary chemotherapy in the treatment of inflammatory breast carcinoma: a study of 230 cases from the Institut Gustave Roussy. *J. Clin. Oncol.*, **4**, 1765–1771.

SCHAFER, P., ALBERTO, P., FORNI, M., OBRADOVIC, D., PIPARD, G. & KRAUER, F. (1987). Surgery as part of a combined modality approach for inflammatory breast carcinoma. *Cancer*, **59**, 1063–1067.

SWAIN, S.M. & LIPPMAN, M.E. (1989). Treatment of patients with inflammatory breast cancer. In *Important advances in Oncology*, De Vita, V., Hellman, S., Rosenberg, S. (eds) J.B. Lippincott Company: Philadelphia, 129–150.

SWAIN, S., SORACE, R., BAGLEY, C., DANFORTH, D., BADER, J., WESLEY, M., STEINBERG, S. & LIPPMAN, M. (1987). Neoadjuvant chemotherapy in the combined modality approach of locally advanced non metastatic breast cancer. *Cancer Res.*, **47**, 3889–3894.

THOMS, W.W., MCNEESE, M.D., FLETCHER, G.H., BUZDAR, A.U., SINGLETARY, E. & OSWALD, M.J. (1990). Multimodal treatment for inflammatory breast cancer. *Int. J. Rad. Oncol. Biol. Phys.*, **17**, 739–745.

WISEMAN, C., JESSUP, J.M., SMITH, T.L., HERSH, E., BOWEN, J. & BLUMENSHEIN, G. (1982). Inflammatory breast cancer treated with surgery, chemotherapy and allogenic tumor cell/BCG immunotherapy. *Cancer*, **49**, 1266–1271.